# Gray wolves in an anthropogenic context on a small island in prehistoric Scandinavia

**DOI:** 10.1073/pnas.2421759122

**Published:** 2025-11-24

**Authors:** Linus Girdland-Flink, Anders Bergström, Jan Storå, Erik Ersmark, Jan Apel, Maja Krzewińska, Love Dalén, Anders Götherström, Pontus Skoglund

**Affiliations:** ^a^Department of Archaeology, University of Aberdeen, Aberdeen AB24 3UF, United Kingdom; ^b^School of Biological and Environmental Sciences, Liverpool John Moores University, Liverpool L3 3AF, United Kingdom; ^c^School of Biological Sciences, University of East Anglia, Norwich NR4 7TJ, United Kingdom; ^d^Ancient Genomics Laboratory, The Francis Crick Institute, London NW1 1AT, United Kingdom; ^e^Department of Archaeology and Classical Studies, Stockholm University, Stockholm SE-106 91, Sweden; ^f^Centre for Palaeogenetics, Stockholm SE-106 91, Sweden; ^g^Department of Zoology, Stockholm University, Stockholm SE-106 91, Sweden; ^h^Department of Bioinformatics and Genetics, Swedish Museum of Natural History, Stockholm SE-104 05, Sweden

**Keywords:** ancient DNA, gray wolves, dogs, bronze age, domestication

## Abstract

Dogs were domesticated at least once from a yet-unidentified wolf population at least ~15,000 y ago. However, how domestication took place is a topic of ongoing debate, and the ability of human groups to manage wolves in their communities during early stages of domestication is poorly understood. Here, we report multiproxy data from two canids excavated from Late Neolithic and Bronze Age contexts in the Stora Förvar cave on the island of Stora Karlsö in the Baltic Sea. The island is small (2.5 sq km) and, like the neighboring island of Gotland, carries no endemic populations of terrestrial mammals. Instead, the current consensus is that human introductions account for some mammal fauna on Gotland, and for the majority of that on Stora Karlsö. Genome-wide data show that the two canids have ancestry indistinguishable from Eurasian wolves, with no shared ancestry with domestic dogs of the *Canis familiaris* lineage. Their genome-wide heterozygosity is lower than that observed in 72 previously published ancient wolf genomes, and instead comparable to dogs. Stable isotope data (δ^13^C and δ^15^N) reveals a diet rich in marine protein, which is consistent with habitation alongside the human groups who used Stora Karlsö as a seal-hunting, fowling, and sea fishing station, and in the Bronze Age probably also for grazing. Skeletal size is at the lower end of wolf variability, and one individual shows advanced pathology consistent with reduced mobility. While other scenarios are possible, a parsimonious explanation is that these wolves were brought to the island by humans and were possibly under human control.

## Significance

Wolves, the wild ancestor of dogs, are the only large carnivores that have undergone domestication by humans. Yet, it remains unclear if this process took place via direct and deliberate human control of wild wolves or if wolf populations gradually adapted to the human niche. Here, we report two canid individuals with gray wolf genetic ancestry excavated from a human archaeological site on a small isolated island in the Baltic Sea dated to between three and 5,000 y ago. The remote island location in combination with the anthropogenic burial context, low genome-wide heterozygosity, marine-rich diet, and small size, are all consistent with a scenario in which these individuals were under human control, but other explanations are also possible.

The dog domestication process is unique in having occurred in the Paleolithic era, predating all other domesticates. However, where and how many times this process took place remains unknown. A central focus in the debate has revolved around possible pathways to domestication (1, 2), including the “commensal pathway”—which posits that wolves effectively self-tamed by settling in human-adjacent niches as an adaptive strategy, and the “direct pathway”—which posits deliberate rearing of wolf pups (3), who, unlike juveniles and adult wolves, are amenable to taming if hand-reared from a very early age (4). One of the main issues with resolving these questions is the difficulty in identifying the earliest (or incipient) stages of domestication in the zooarchaeological record (5, 6). Unlike large-scale domestication of herd animals, which affords the compilation of large archaeological time-series data from which demographic signatures of domestication can be documented (7, 8), no dog remains clearly dating to the initial stages of domestication are currently known. Early indicators of domestication include a close and sustained association with humans or archaeological sites, dietary shifts due to provisioning or human control, reduced body size, and declines in genetic diversity (e.g., lower heterozygosity), although these signatures may be subtle or incompletely represented in small, early-stage populations even when under human control (7, 8).

Here, we leverage the ability of genomics to distinguish dogs and wolves by generating genome-wide data from two canid remains (G.7 and G.11) excavated from the Stora Förvar cave on the island of Stora Karlsö in the Baltic Sea. The site has yielded three cranial and several postcranial bone elements of at least one domestic dog, but also at least one individual possessing size and morphological features interpreted as consistent with a wolf reared under human control (9). Since more recent research has shown that distinguishing wolves from early domestic dogs based on morphology alone is difficult (10), the initial interpretation poses an interesting question—were wolves under human control in prehistoric Scandinavia? Although we were unable to locate the cranial elements described in 1926, we identified a distal humerus of an adult canid from section I.5–which is a domestic dog from which genomic data was published previously (11)—and several metapodia of a juvenile canid from section G.7 (directly dated to the Middle Neolithic (4804-4601 cal BP, 2σ) (Table 1 and *SI Appendix*, Table S1). Additionally, we found a previously undescribed distal humerus from an adult canid from section G.11, which we directly dated to the Bronze Age (3304-3094 cal BP, 2σ) (Table 1). We also obtained osteometrics (Bd) and assessed diet via δ^13^C and δ^15^N isotopes.

**Table 1.** Table with sample information

Find location/element	G.7 metapodia	G.11 humerus	I.5 humerus
ID, Radiocarbon date	Beta-440526	Beta-440525	Beta-440527
Conventional radiocarbon age	4,290 ± 30 BP	3,140 ± 30 BP	3,680 ± 30 BP
Calibrated Dates (95.4% probability) with reservoir correction 162 ± 30 y	4804-4601 cal BP/2860-2640 cal BCE	3304-3094 cal BP/1360-1140 cal BCE	4141-3910 cal BP/2200-1960 cal BCE
δ^13^C (‰)	−14.1	−17.2	−20.3
δ^15^N (‰)	11	13	8.3
CN	+3.2	+3.2	+3.2
Wt %C	+42.88	+42.80	+42.28
Wt %N	+15.49	+15.65	+15.58

**Fig. 1.**
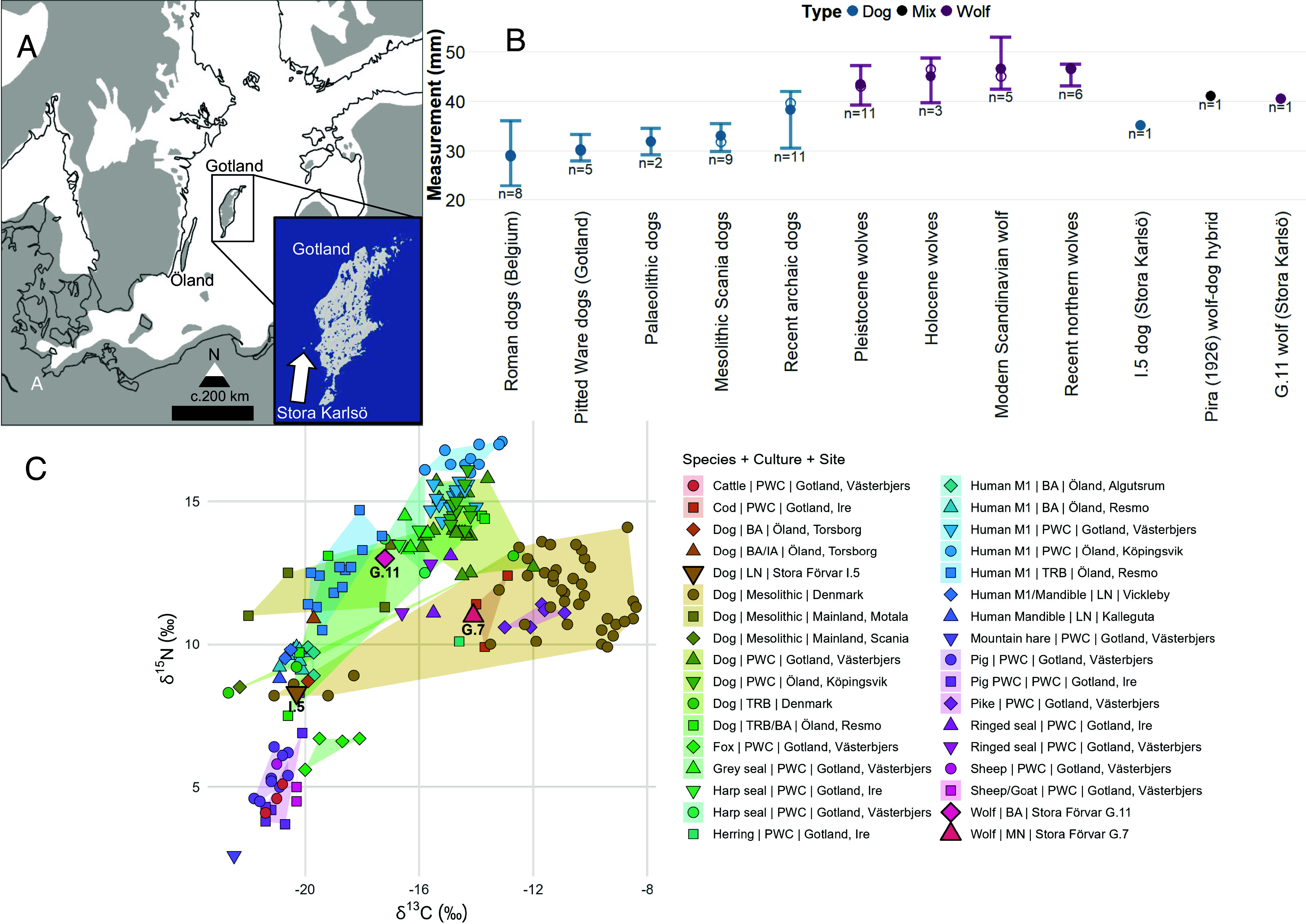
(*A*) Map showing the location of Gotland, Stora Karlsö and Öland in the Baltic Sea. The gray area shows the reconstructed shoreline approximately 9,000 cal BP (12). (*B*) A range plot of humeral greatest distal breadth (Bd) in modern and ancient dogs and wolves, including a wolf-dog hybrid from the comparative dataset used by Pira (9) (*SI Appendix*, Table S3). (*C*) δ^13^C and δ^15^N stable isotope values of a representative sample of dogs, humans, and fauna from approximately contemporaneous archaeological sites on Gotland, Stora Karlsö and Öland, and Mesolithic dogs from mainland Sweden and Denmark (*SI Appendix*, Table S4). PWC=Pitted Ware Culture, TRB=Trichterbecherkultur or Funnel Beaker Culture (Early to Middle Neolithic), MN = Middle Neolithic, LN = Late Neolithic, BA = Bronze Age. See *SI Appendix*, Table S1 for archaeological periods and associated calendar years.

The archaeological context of the Stora Förvar cave provides a compelling set of circumstances for testing the hypothesis of wolves under some degree of human control (9). Stora Karlsö is a small, round-shaped limestone island (ca 2.5 sq km) located about 5 km west of the larger island of Gotland and around 80 km east of mainland Sweden (Fig. 1*A*). The islands were never connected to mainland Scandinavia, and consequently, mammals currently inhabiting the larger island of Gotland such as hares, foxes, wild or feral pigs (now extinct), and hedgehogs were never native but likely introduced by humans (12). Importantly, as there are no bones of wolf or of large canids in any of the faunal assemblages from Gotlandic Neolithic or Bronze Age sites (13, 14), the presence of wolves at Stora Förvar would imply that they were brought to the island by humans. Excavations of the cave between 1888 and 1893 revealed a rich archive of material culture and faunal remains in up to 4 m thick cultural layers in the c. 25 m deep cave and spanning around 8,000 y (9, 12). The cultural layers in the Stora Förvar cave are entirely anthropogenic and the assemblage comprises thousands of skeletal fragments—all likely representing food waste—from numerous species, dominated by seals. Domestic or commensal animals occur in the upper layers, including cattle, sheep, goat, pig, horse, but also mice, and fox (9). Scattered human remains were also found in the layers (15), as was a domestic dog (I.5) (11), and the wolves hypothesized to be under human control (9).

## Results and Discussion

**Fig. 2.**
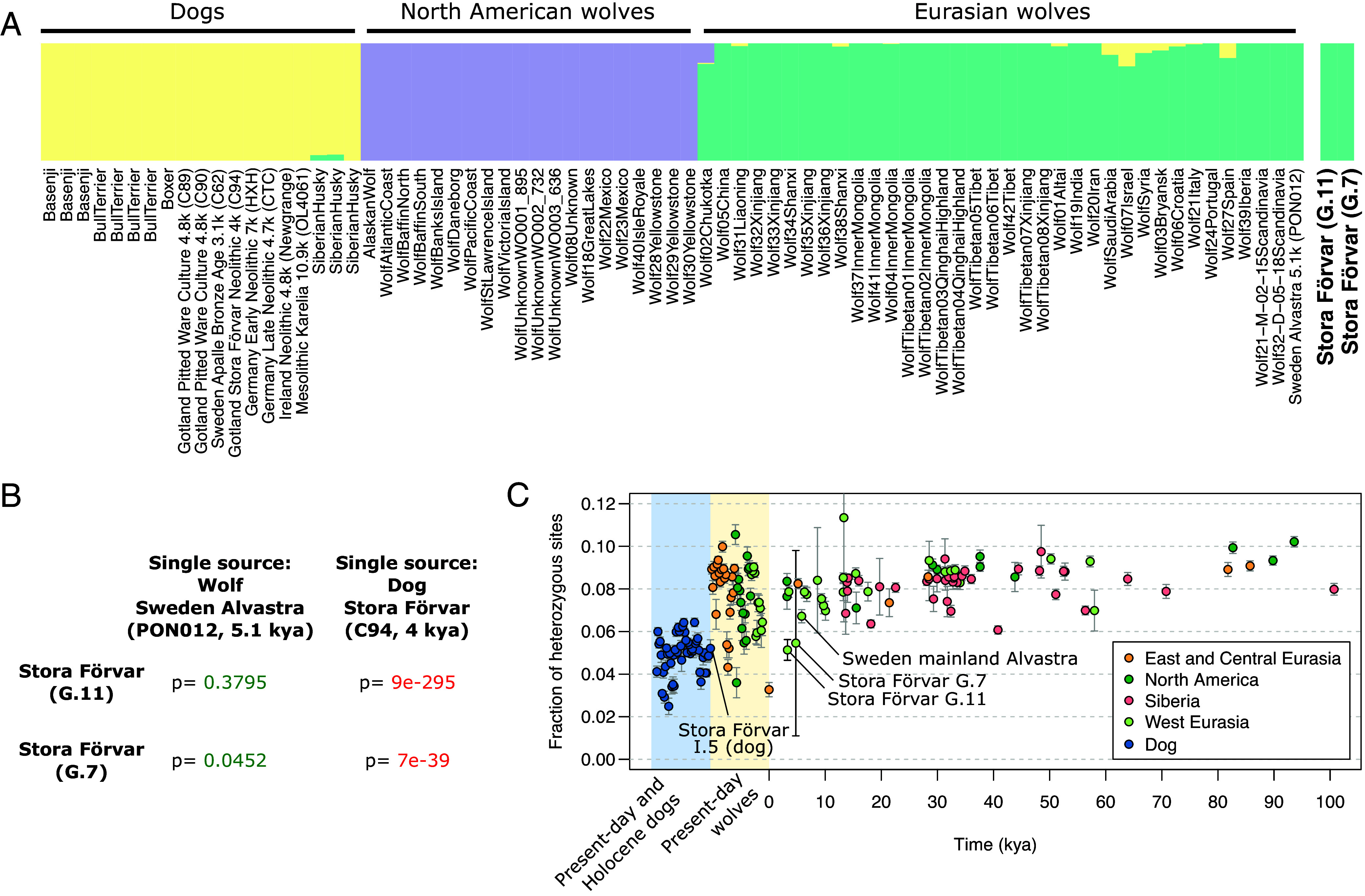
Genetic identities of the Stora Förvar wolves. (*A*) ADMIXTURE clustering of a dataset containing dogs, wolves, and the G.11 and G.7 wolves. (*B*) *qpWave/qpAdm* results for the Stora Förvar wolves. The best-fitting models are single-source models using a 5.1k y-old Swedish wolf as the source, to the exclusion of dogs. (*C*) Heterozygosity estimates for the Stora Förvar wolves, in the context of Late Pleistocene, Holocene, and present-day wolves, as well as Holocene and present-day dogs. Error bars correspond to ±2 SE.

We sequenced the genomes of G.11 and G.7 to 0.1× and 0.008× depth coverage, respectively, to test whether they were dogs, wolves, or hybrids (*SI Appendix*, Table S2). While low coverage, the genetic bottleneck that is hypothesized to be shared by all modern and ancient domestic dogs (16) means that distinguishing wolves from the domestic dog lineage, at least in the Holocene, is usually possible even with limited data. The model-based clustering method *ADMIXTURE* (17) estimates that both individuals entirely carried wolf-like ancestry (Fig. 2*A*). Confirming this more formally using *qpWave*/*qpAdm* (18), the best fit for both G.11 (*P* = 0.37) and G.7 (*P* = 0.045) is a single-source model using a ca. 5,100-y-old wolf genome (2.1x coverage) from Alvastra on mainland Scandinavia as the best representative for their ancestry among tested sources (16) (Fig. 2*B*). Importantly, our *qpAdm* models include the previously identified ~4,000-y-old I.5 dog from Stora Karlsö (11) as an outgroup, revealing no indication of admixture with local dogs. If we estimate an upper limit on the proportion of *possible* dog ancestry by force-fitting a two-way model that includes the I.5 dog as a source, we obtain point estimates of 4.4% (G.11, SE 4.7%) and 16.5% (G.7, SE 9.2%) dog ancestry. While we thus cannot formally exclude minority proportions of dog-related ancestry, there is no statistically significant evidence that these individuals carried it, and the most parsimonious model is that they carried exclusively wolf ancestry.

We furthermore confirm original observations of relatively small size of these wolves (9), as the greatest distal breadth (Bd) of the G.11 humerus measures 40.5 mm (Fig. 1*B* and *SI Appendix*, Table S3), which is larger than any domestic Mesolithic or contemporaneous Pitted Ware Culture (PWC) dogs on Gotland but smaller than a sample of modern Scandinavian wolves (14) and at the lower end of the size range of Pleistocene and Holocene wolves (Fig. 1*B* and *SI Appendix*, Table S3). These data are consistent with G.11 representing a small wolf, even when accounting for sexual dimorphism (G.11 is a female).

We then assessed evidence for inbreeding, which is a common consequence of longer-term human management of animals. We find that G.11 had a heterozygosity level that is lower than that observed in any other ancient wolf in a world-wide dataset of 72 genomes from the Late Pleistocene and Holocene, including the roughly contemporaneous Alvastra wolf (16) (Fig. 2*C*). In principle, such a pattern would be expected under a scenario of multigenerational reproductive control of a small wolf population, consistent with Pira’s original hypothesis (9), but such lower genetic diversity could also arise from a restricted effective population size in wild populations on a short time scale. Indeed, some present-day wolves display heterozygosity levels as low, or lower, than the G.11 genome, likely reflecting bottlenecks and isolation caused by recent human encroachment and habitat loss.

Last, the δ^13^C and δ^15^N isotopes obtained from C14 dating show that both G.7 and G.11 had a diet enriched in marine protein, unlike the I.5 dog whose diet primarily was based on terrestrial resources, consistent with the diet of Late Neolithic and Bronze Age dogs and humans from Öland (Fig. 1*C* and *SI Appendix*, Table S4) (19, 20). The diet of G.7 indicates a high proportion of low trophic level marine protein such as fish, similar to a subset of Mesolithic dogs from Denmark (21), and some contemporaneous PWC dogs from Gotland (Fig. 1*C*). Sustained access to fish or other low trophic level marine resources suggests a potential dependency on humans, as independently acquiring such foods is challenging for dogs or wolves (21), particularly in the absence of seasonally abundant fish like salmon (22, 23). However, most dogs and humans from PWC Gotland and Öland show higher δ^15^N values consistent with a greater reliance on higher trophic level marine protein such as seals (Fig. 1*C* and *SI Appendix*, Table S4). G.11 likely had a mixed terrestrial and marine diet similar to some Mesolithic dogs from mainland Sweden and Neolithic humans from Öland but is an outlier compared to most Bronze Age dogs and humans (Fig. 1*C*; *SI Appendix*, Table S4).

We note that while marine diets in wolves have been observed previously, most notably in the Alaska and British Columbia region in North America, these wolves tend to scavenge in archipelagos and along coast lines (22, 23), and to our knowledge there is no zooarchaeological or isotopic evidence of resident or seasonally marine-scavenging wolves in prehistoric Scandinavia. While crossing ice remains a possibility, the minimum distance to the mainland (~80 km) would be at the extreme upper edge of what has been documented or deemed plausible (24). The G.11 individual also exhibits pathological lesions in the humerus, above the distal trochlea in the olecranon fossa, which must have affected the individual for a prolonged period and likely reduced its mobility. It therefore seems unlikely that the wolves on Stora Karlsö represent a naturally dispersed, marine-adapted scavenging population.

While the presence of wolves in human contexts is rare in the archaeological record, it is not without precedent. Across Eurasia, there are instances where wolves appear to have been incorporated into prehistoric human societies, not merely as wild fauna, but as animals with symbolic, ritual, and possibly practical meaning (25). It is possible that the bone remains came from hides used and discarded by humans, but this would be expected to lead to a nonrandom representation of skeletal elements, particularly the peripheral limb parts like toe bones, and metapodia. While metapodia (G.7) are commonly found in hides, the humerus (G11) and cranial elements (9) would not be expected to be part of hides. However, this hypothesis fails to explain the marine-rich diet which suggests longer-term adaptation to a marine environment.

## Conclusion

The co-occurrence of geographic isolation in a firmly anthropogenic archaeological context, unusual marine-enriched diet, small size, and reduced heterozygosity, all of which are often considered domestication markers (7, 8), suggests the possibility of prehistoric human control of wolves. This finding implies that similar processes may have taken place elsewhere but in ways that may have left a more ambiguous archaeological record. However, other scenarios such as natural dispersal cannot be excluded. Our results provide evidence that extends the discourse about past human–wolf interactions and relationships.

## Materials and Methods

The canid bones are held and curated by National Historical Museums in Stockholm, Sweden. We obtained relevant permissions for destructive testing prior to commencing research. Ancient DNA lab work was performed in dedicated clean-room facilities at the Archaeological Research Laboratory and Centre for Palaeogenetics at Stockholm University, Sweden. DNA extraction, double-stranded library construction, and UDG (uracil DNA glycosylase) treatment was performed as previously described (11). Bioinformatics processing was also as previously described (11).In short, we compiled a set of 65,541,655 single-nucleotide variants, at which we sampled a random allele in the ancient genomes if covered by at least one read ≥35 bp with mapping quality ≥20, and a base quality of ≥30. The sequencing data produced in the study are archived in the European Nucleotide Archive, study accession PRJEB81829. AMS radiocarbon dating was carried out by Beta Analytic Inc. Dates were calibrated using OxCal v4.4.4 (19) with the atmospheric data in IntCal20 (20). For G.7 and G.11, we adjusted for the marine Baltic reservoir age offset R(t) using an empirically determined value of 162 ± 30 y (26). δ^13^C and δ^15^N stable isotope values were reported by Beta Analytic as part of the AMS radiocarbon dating. Additional information about the comparative datasets compiled for Fig. 1 *B* and *C* are presented in *SI Appendix*, Tables S3 and S4.

## Supplementary Material

Appendix 01 (PDF)

## Data Availability

DNA sequence data have been deposited in European Nucleotide Archive (PRJEB81829) (27).
