# Supplementary material for: Gray wolves in an anthropogenic context on a small island in prehistoric Scandinavia
Source: Proc Natl Acad Sci U S A. 2025 Nov 24;122(48):e2421759122. doi: 10.1073/pnas.2421759122 (PMC12684923; doi:10.1073/pnas.2421759122)
Supplement: Supplementary file 1 — Appendix 01 (PDF) [file pnas.2421759122.sapp.pdf]

PNAS

Supporting Information

Ancient DNA extended methods

Paired-end reads obtained for the G.11 and G.7 samples were merged and trimmed for adaptor using SeqPrep (https://github.com/jstjohn/SeqPrep), discarding read pairs that could not be merged. BWA aln(1) (v.0.7.17) with permissive parameters (-l 16500 -n 0.01 -o 2) was used to map reads to the dog reference genome canFam3.1. Duplicate reads were excluded by keeping one read from any set of that had the same orientation, length and start and end coordinates. We used PMDtools (https://github.com/pontussk/PMDtools)(2) to verify that both genomes displayed ancient DNA damage at CpG sites, as expected given that the libraries were UDG-treated. Pseudo-haploid genotypes for the G.11 and G.7 genomes were then obtained from the read alignments using htsbox pileup r345 (https://github.com/lh3/htsbox), sampling a random allele with a base quality of ≥30, from reads with mapping quality ≥20 and length of ≥35. These genotypes were then merged into a previously described dataset(3) of ancient and present-day wolf, dog, coyote and other canid genomes compiled from multiple previous studies, containing genotype calls at 65,541,655 single-nucleotide variants. This dataset includes genomes from studies archived under the following BioProject accessions: PRJNA448733(4), PRJCA000335(5), PRJEB20635(6), PRJNA496590(7), PRJNA494815(8), PRJEB42199(3), PRJEB38079(9), PRJEB7788(10), PRJEB13070(11), PRJNA319283(12), PRJEB22026(13), PRJNA608847(14), PRJEB39580(15).

Model-based clustering was performed using ADMIXTURE v1.3.0 (16), on a subsetted version of the dataset containing 77 individuals — 7,195,896 transversion variants remained polymorphic in this subsetted dataset, and were used for the ADMIXTURE analyses. *qpWave*/*qpAdm (17)* models were fit using ADMIXTOOLS (v5.0) (18) applying the “allsnps: YES” option, using all 19,299,899 transversion variants in the dataset. Heterozygosity was estimated in individual genomes by sampling exactly two reads at each of 1,250,173 sites ascertained as heterozygous in a present-day coyote genome (sample Coyote01 from California, BioSample accession SAMN02921301), and quantifying the fraction of those sites at which the two reads displayed different alleles (ignoring reads displaying alleles other than the two observed in the coyote). Standard errors were obtained by block jackknifing across the 38 chromosomes.

Isotope extended methods

$\delta^{13}C$ and $\delta^{15}N$ values from AMS radiocarbon dating of specimens I.5, G.7, and G.11 were integrated into a larger comparative dataset (Table S4) containing isotopic data

from humans, dogs, and fauna across archaeological sites in Öland, Gotland, mainland Sweden, and Denmark. The comparative samples represent three dietary categories: individuals with high proportions of low-trophic marine protein, others with high proportions of higher-trophic marine proteins, and those with primarily terrestrial diets, including dogs, humans, cattle, pigs, and foxes (Fig 1C, Table S3).

The dataset spans from the Mesolithic period—primarily its later phase, including Ertebølle forager dogs (5400–4000 cal. BCE) from Denmark—through the Early-Middle Neolithic TRB (Funnel Beaker Culture) and PWC (Pitted Ware Culture) periods, extending to the Late Neolithic and Bronze Age (Table S1, Table S4). While not exhaustive, these isotope analyses provide comparative context for interpreting the likely diets of I.5, G.7, and G.11, all of which exhibit $\delta^{13}C$ and $\delta^{15}N$ profiles closely matching several dogs and humans in the comparative dataset.

Extended osteometric analysis

The dataset includes measurements of Mesolithic dogs from Scania ((19, 20)), PWC dogs from Gotland (housed at Stockholm University's Osteologiska Research Laboratory), modern Scandinavian wolves from the Natural History Museum in Stockholm, and specimens I.5 (dog) and G.11 (wolf). Data from a previous study (21) is compiled from multiple publications and encompasses seven groups of ancient and modern canids. Pleistocene wolves include specimens from Belgium, France, and a Russian Far East cave. Recent northern Eurasian wolves come from Sweden and Russia. Holocene wolves span from postglacial Denmark (containing both the smallest and largest individuals) to a Neolithic Siberian specimen and a Roman-period Belgian wolf. Early domesticated dogs derive from Upper Palaeolithic sites in Spain and France. Roman dogs represent Belgian specimens from two archaeological sites. Recent archaic dogs include samples from Siberia, Sakhalin, and Greenland. We also included a wolf-dog hybrid presented in (22) during the initial analyses of the Stora Förvar canids.

Supplementary Tables

Table S1. Archaeological periods and their approximate dates following ref. 25.

**Period**	**Date range (cal BCE)**	**Date range (cal BP)**
Mesolithic	8200–4000	10150–5950
Early Neolithic	4000–3300	5950–5250
Middle Neolithic	3300–2300	5250–4250
Late Neolithic	2300–1800	4250–3750
Early Bronze Age	1800–1100	3750–3050
Late Bronze Age	1100–500	3050–2450

Table S2. Properties of genome sequencing data

Sample	G.11	G.7
Library strandedness	Double-stranded	Double-stranded
Damage treatment	UDG	UDG
Fraction of first 5' Cs in CpG context showing deamination	0.187	0.260
Number of reads used for analyses (length ≥ 35bp, MQ ≥ 20)	7,653,930	369,530
Average length of reads used in analyses (length ≥ 35bp, MQ ≥ 20)	54.8	47.2
Average coverage (x-fold)	0.1	0.008
Transversion SNPs covered in full dataset (out of 19,299,899)	1,518,813 (7.9%)	131,098 (0.7%)
Transversion SNPs covered in ADMIXTURE dataset (out of 7,195,896)	559,237 (7.8%)	47,983 (0.7%)
Transversion SNPs used in heterozygosity estimates, covered by least two reads (out of 1,250,173)	10,477 (0.8%)	110 (0.009%)
Fraction of sites displaying heterozygosity	0.051350509	0.054545364
Standard error on fraction of sites displaying heterozygosity	0.002434	0.021762

Table S3. Size measurements of distal humeri used for Figure 1B (20–23).

Group	Type	N	Min	Mean	Median	Max	SD	Reference
Palaeolithic dogs	Dog	2	29.2	31.9	31.9	34.5	3.75	(21)
Roman dogs (Belgium)	Dog	8	22.9	29.1	28.9	36.1	4.25	(21)
Recent archaic dogs	Dog	11	30.5	38.28	39.6	42	3.44	(21)
Pleistocene wolves	Wolf	11	39.2	43.46	43	47.2	2.97	(21)
Holocene wolves	Wolf	3	39.7	45	46.5	48.8	4.73	(21)
Recent northern wolves	Wolf	6	43.1	46.68	46.55	47.5	1.65	(21)
Mesolithic Scania dogs	Dog	9	29.90	33.03	31.80	35.50	2.28	(23)
Pitted Ware dogs (Gotland)	Dog	5	27.93	30.29	30.05	33.30	2.01	This study
Modern Scandinavian wolf	Wolf	5	42.49	46.55	45.05	53.00	4.14	(20)
I.5 dog (Stora Karlsö)	Dog	1	-	35.1	-	-	-	This study
G.11 wolf (Stora Karlsö)	Wolf	1	-	40.50	-	-	-	This study
Pira (1926) wolf-dog hybrid	Mix	1	-	41.00	-	-	-	(22)

Table S4. $\delta^{13}C$ and $\delta^{15}N$ values used for Figure 1C (24–27) .

Species	Culture	Sample group	$\delta^{13}C$ (‰)	$\delta^{15}N$ (‰)	Reference
Cattle	PWC	Gotland, Västerbjers	-20.8	5.1	(24)
Cattle	PWC	Gotland, Västerbjers	-21.0	4.6	(24)
Cattle	PWC	Gotland, Västerbjers	-21.4	4.1	(24)
Cod	PWC	Gotland, Ire	-13.7	9.9	(24)
Cod	PWC	Gotland, Ire	-14.0	11.4	(24)
Cod	PWC	Gotland, Ire	-12.9	12.4	(24)
Dog	PWC	Gotland, Västerbjers	-12.0	12.7	(24)
Dog	PWC	Gotland, Västerbjers	-15.8	13.8	(24)
Dog	PWC	Gotland, Västerbjers	-14.6	13.9	(24)
Dog	PWC	Gotland, Västerbjers	-14.2	13.8	(24)
Dog	PWC	Gotland, Västerbjers	-14.3	13.8	(24)
Dog	PWC	Gotland, Västerbjers	-14.5	13.9	(24)
Dog	PWC	Gotland, Västerbjers	-14.2	12.5	(24)
Dog	PWC	Gotland, Västerbjers	-15.3	15.1	(24)
Dog	PWC	Gotland, Västerbjers	-14.5	12.4	(24)
Dog	PWC	Gotland, Västerbjers	-14.4	13.9	(24)
Dog	PWC	Gotland, Västerbjers	-14.6	15.3	(24)
Dog	PWC	Gotland, Västerbjers	-14.8	14.0	(24)
Dog	PWC	Gotland, Västerbjers	-15.4	15.7	(24)
Dog	PWC	Gotland, Västerbjers	-13.6	15.8	(24)
Dog	PWC	Gotland, Västerbjers	-15.5	14.0	(24)
Dog	PWC	Gotland, Västerbjers	-14.3	15.3	(24)
Dog	PWC	Gotland, Västerbjers	-15.9	13.4	(24)
Dog	PWC	Gotland, Västerbjers	-14.7	13.9	(24)

Species	Culture	Sample group	δ^{13}C (‰)	δ^{15}N (‰)	Reference
Dog	PWC	Gotland, Västerbjers	-14.5	15.3	(24)
Dog	PWC	Öland, Köpingsvik	-14.8	14.5	(25)
Dog	PWC	Öland, Köpingsvik	-14.2	14.7	(25)
Dog	PWC	Öland, Köpingsvik	-14.9	14.4	(25)
Dog	PWC	Öland, Köpingsvik	-14.4	14.0	(25)
Dog	PWC	Öland, Köpingsvik	-14.7	14.7	(25)
Dog	PWC	Öland, Köpingsvik	-14.4	15.6	(25)
Dog	PWC	Öland, Köpingsvik	-14.9	14.2	(25)
Dog	PWC	Öland, Köpingsvik	-14.2	14.5	(25)
Dog	PWC	Öland, Köpingsvik	-14.8	14.9	(25)
Dog	PWC	Öland, Köpingsvik	-14.3	16.1	(25)
Dog	PWC	Öland, Köpingsvik	-14.7	15.0	(25)
Dog	TRB/BA	Öland, Resmo	-20.6	7.5	(25)
Dog	TRB/BA	Öland, Resmo	-13.8	14.5	(25)
Dog	TRB/BA	Öland, Resmo	-19.2	13.1	(25)
Dog	TRB/BA	Öland, Resmo	-13.7	14.4	(25)
Dog	TRB/BA	Öland, Resmo	-20.2	9.7	(25)
Dog	BA	Öland, Torsborg	-19.9	8.7	(25)
Dog	BA/IA	Öland, Torsborg	-19.7	10.9	(25)
Dog	Mesolithic	Mainland, Scania	-22.3	8.5	(28)
Dog	Mesolithic	Mainland, Motala	-20.6	12.5	(28)
Dog	Mesolithic	Mainland, Motala	-17.2	11.3	(28)
Dog	Mesolithic	Mainland, Motala	-17.0	13.5	(28)
Dog	Mesolithic	Mainland, Motala	-22.0	11.0	(28)
Dog	Mesolithic	Denmark	−8.5	11.5	(26)
Dog	Mesolithic	Denmark	−8.4	11.3	(26)

Species	Culture	Sample group	δ13C (‰)	δ15N (‰)	Reference
Dog	Mesolithic	Denmark	−10.9	11.8	(26)
Dog	Mesolithic	Denmark	−11.7	13.6	(26)
Dog	Mesolithic	Denmark	−11.4	11.3	(26)
Dog	Mesolithic	Denmark	−11.0	11.9	(26)
Dog	Mesolithic	Denmark	−21.1	8.2	(26)
Dog	Mesolithic	Denmark	−12.5	13.4	(26)
Dog	Mesolithic	Denmark	−11.4	13.5	(26)
Dog	Mesolithic	Denmark	−10.6	12.3	(26)
Dog	Mesolithic	Denmark	−10.2	13.0	(26)
Dog	Mesolithic	Denmark	−10.2	11.7	(26)
Dog	Mesolithic	Denmark	−10.3	12.1	(26)
Dog	Mesolithic	Denmark	−11.3	12.2	(26)
Dog	Mesolithic	Denmark	−11.6	12.5	(26)
Dog	Mesolithic	Denmark	−12.3	10.7	(26)
Dog	Mesolithic	Denmark	−20.4	8.6	(26)
Dog	Mesolithic	Denmark	−13.5	10.0	(26)
Dog	Mesolithic	Denmark	−11.9	10.1	(26)
Dog	Mesolithic	Denmark	−13.2	11.9	(26)
Dog	Mesolithic	Denmark	−18.3	8.9	(26)
Dog	Mesolithic	Denmark	−10.4	11.4	(26)
Dog	Mesolithic	Denmark	−10.3	13.3	(26)
Dog	Mesolithic	Denmark	−10.0	12.7	(26)
Dog	Mesolithic	Denmark	−11.2	12.6	(26)
Dog	Mesolithic	Denmark	−10.5	13.5	(26)
Dog	Mesolithic	Denmark	−8.7	14.1	(26)
Dog	Mesolithic	Denmark	−11.3	12.4	(26)

Species	Culture	Sample group	δ13C (‰)	δ15N (‰)	Reference
Dog	Mesolithic	Denmark	−9.4	10.9	(26)
Dog	Mesolithic	Denmark	−12.2	13.0	(26)
Dog	Mesolithic	Denmark	−19.2	8.2	(26)
Dog	Mesolithic	Denmark	−12.2	12.4	(26)
Dog	Mesolithic	Denmark	−9.6	10.0	(26)
Dog	Mesolithic	Denmark	−9.2	10.3	(26)
Dog	Mesolithic	Denmark	−9.4	9.9	(26)
Dog	Mesolithic	Denmark	−9.1	10.7	(26)
Dog	Mesolithic	Denmark	−8.5	10.9	(26)
Dog	Mesolithic	Denmark	−9.6	10.5	(26)
Dog	Mesolithic	Denmark	−8.8	10.8	(26)
Dog	Mesolithic	Denmark	−10.8	10.7	(26)
Dog	Mesolithic	Denmark	−10.9	11.5	(26)
Dog	TRB	Denmark	−20.3	9.2	(26)
Dog	TRB	Denmark	−22.7	8.3	(26)
Dog	TRB	Denmark	−12.7	13.1	(26)
Dog	Neolithic	Stora Förvar J5	-20.3	8.3	This study
Fox	PWC	Gotland, Västerbjers	-18.1	6.7	(24)
Fox	PWC	Gotland, Västerbjers	-18.7	6.6	(24)
Fox	PWC	Gotland, Västerbjers	-19.5	6.7	(24)
Fox	PWC	Gotland, Västerbjers	-20.0	5.6	(24)
Grey seal	PWC	Gotland, Västerbjers	-16.4	13.3	(24)
Grey seal	PWC	Gotland, Västerbjers	-15.7	13.9	(24)
Grey seal	PWC	Gotland, Västerbjers	-16.5	14.5	(24)
Grey seal	PWC	Gotland, Västerbjers	-16.3	13.4	(24)
Harp seal	PWC	Gotland, Västerbjers	-15.8	12.5	(24)

Species	Culture	Sample group	$\delta^{13}C$ (‰)	$\delta^{15}N$ (‰)	Reference
Harp seal	PWC	Gotland, Västerbjers	-17.2	13.7	(24)
Harp seal	PWC	Gotland, Västerbjers	-15.8	16.1	(24)
Harp seal	PWC	Gotland, Ire	-16.7	13.5	(24)
Harp seal	PWC	Gotland, Ire	-16.0	14.0	(24)
Herring	PWC	Gotland, Ire	-14.6	10.1	(24)
Human M1	PWC	Gotland, Västerbjers	-14.6	15.7	(24)
Human M1	PWC	Gotland, Västerbjers	-15.6	14.7	(24)
Human M1	PWC	Gotland, Västerbjers	-15.3	14.9	(24)
Human M1	PWC	Gotland, Västerbjers	-14.8	15.2	(24)
Human M1	PWC	Gotland, Västerbjers	-15.4	15.1	(24)
Human M1	PWC	Gotland, Västerbjers	-14.7	15.3	(24)
Human M1	PWC	Gotland, Västerbjers	-15.5	15.6	(24)
Human M1	PWC	Gotland, Västerbjers	-14.9	14.8	(24)
Human M1	PWC	Gotland, Västerbjers	-14.7	15.4	(24)
Human M1	PWC	Gotland, Västerbjers	-14	14.8	(24)
Human M1	PWC	Gotland, Västerbjers	-14.3	15.6	(24)
Human M1	PWC	Gotland, Västerbjers	-14.4	15.4	(24)
Human M1	PWC	Gotland, Västerbjers	-15.2	14.3	(24)
Human M1	PWC	Öland, Köpingsvik	-13.9	17.0	(25)
Human M1	PWC	Öland, Köpingsvik	-14.4	16.3	(25)
Human M1	PWC	Öland, Köpingsvik	-13.1	17.1	(25)
Human M1	PWC	Öland, Köpingsvik	-14.2	16.5	(25)
Human M1	PWC	Öland, Köpingsvik	-15.1	16.8	(25)
Human M1	PWC	Öland, Köpingsvik	-14.2	16.0	(25)
Human M1	PWC	Öland, Köpingsvik	-14.9	16.3	(25)
Human M1	PWC	Öland, Köpingsvik	-15.8	16.1	(25)

Species	Culture	Sample group	$\delta^{13}C$ (‰)	$\delta^{15}N$ (‰)	Reference
Human M1	PWC	Öland, Köpingsvik	-13.9	16.3	(25)
Human M1	PWC	Öland, Köpingsvik	-13.2	17.0	(25)
Human M1	TRB	Öland, Resmo	-18.6	12.6	(25)
Human M1	TRB	Öland, Resmo	-19.0	11.8	(25)
Human M1	TRB	Öland, Resmo	-19.6	11.3	(25)
Human M1	TRB	Öland, Resmo	-18.8	12.7	(25)
Human M1	TRB	Öland, Resmo	-17.3	13.8	(25)
Human M1	TRB	Öland, Resmo	-19.5	12.4	(25)
Human M1	TRB	Öland, Resmo	-19.8	12.5	(25)
Human M1	TRB	Öland, Resmo	-19.4	10.5	(25)
Human M1	TRB	Öland, Resmo	-18.1	14.7	(25)
Human M1	TRB	Öland, Resmo	-18.4	12.7	(25)
Human M1	BA	Öland, Resmo	-20.4	9.8	(25)
Human M1	BA	Öland, Resmo	-20.3	9.9	(25)
Human M1	TRB	Öland, Resmo	-18.0	13.3	(25)
Human M1	TRB	Öland, Resmo	-18.7	12.0	(25)
Human M1	BA	Öland, Resmo	-20.1	9.1	(25)
Human M1	BA	Öland, Resmo	-20.6	9.6	(25)
Human M1	BA	Öland, Resmo	-20.9	9.2	(25)
Human M1	TRB	Öland, Resmo	-19.9	11.4	(25)
Human M1	BA	Öland, Resmo	-19.9	9.7	(25)
Human M1	BA	Öland, Resmo	-20.2	9.8	(25)
Human M1	BA	Öland, Resmo	-20.2	9.4	(25)
Human M1	BA	Öland, Algutsrum	-19.9	9.9	(25)
Human M1	BA	Öland, Algutsrum	-19.7	8.9	(25)
Human M1	BA	Öland, Algutsrum	-19.7	9.7	(25)

Species	Culture	Sample group	δ^{13}C (‰)	δ^{15}N (‰)	Reference
Human M1	LN	Vickleby	-20.5	9.8	(27)
Human Mandible	LN	Vickleby	-20.7	9.5	(27)
Human Mandible	LN	Kalleguta	-20.9	8.8	(27)
Mountain hare	PWC	Gotland, Västerbjers	-22.5	2.6	(24)
Pig	PWC	Gotland, Västerbjers	-21.8	4.6	(24)
Pig	PWC	Gotland, Västerbjers	-20.6	6.2	(24)
Pig	PWC	Gotland, Västerbjers	-20.9	5.0	(24)
Pig	PWC	Gotland, Västerbjers	-21.6	4.5	(24)
Pig	PWC	Gotland, Västerbjers	-20.8	6.1	(24)
Pig	PWC	Gotland, Västerbjers	-21.1	6.4	(24)
Pig	PWC	Gotland, Västerbjers	-21.2	5.3	(24)
Pig	PWC	Gotland, Västerbjers	-20.6	5.4	(24)
Pig	PWC	Gotland, Västerbjers	-21.2	5.2	(24)
Pig PWC	PWC	Gotland, Ire	-20.1	6.9	(24)
Pig PWC	PWC	Gotland, Ire	-21.2	4.2	(24)
Pig PWC	PWC	Gotland, Ire	-21.4	3.8	(24)
Pig PWC	PWC	Gotland, Ire	-20.7	3.7	(24)
Pig PWC	PWC	Gotland, Ire	-21.4	4.3	(24)
Pike	PWC	Gotland, Västerbjers	-10.9	11.1	(24)
Pike	PWC	Gotland, Västerbjers	-12.1	10.6	(24)
Pike	PWC	Gotland, Västerbjers	-13.0	10.6	(24)
Pike	PWC	Gotland, Västerbjers	-11.7	11.4	(24)
Pike	PWC	Gotland, Västerbjers	-11.6	11.2	(24)
Ringed seal	PWC	Gotland, Ire	-15.5	11.1	(24)
Ringed seal	PWC	Gotland, Ire	-14.9	13.1	(24)
Ringed seal	PWC	Gotland, Västerbjers	-15.6	12.8	(24)

Species	Culture	Sample group	δ^{13}C (‰)	δ^{15}N (‰)	Reference
Ringed seal	PWC	Gotland, Västerbjers	-16.6	11.1	(24)
Sheep	PWC	Gotland, Västerbjers	-21.0	5.8	(24)
Sheep/Goat	PWC	Gotland, Västerbjers	-20.2	8.3	(24)
Sheep/Goat	PWC	Gotland, Västerbjers	-20.3	4.5	(24)
Sheep/Goat	PWC	Gotland, Västerbjers	-20.3	5.0	(24)
Wolf	Neolithic	Stora Förvar G7	-14.1	11	This study
Wolf	BA	Stora Förvar G11	-17.2	13	This study

References

1. H. Li, R. Durbin, Fast and accurate short read alignment with Burrows-Wheeler transform. *Bioinformatics* **25**, 1754–1760 (2009).
2. P. Skoglund, *et al.*, Separating endogenous ancient DNA from modern day contamination in a Siberian Neandertal. *Proc. Natl. Acad. Sci. U. S. A.* **111**, 2229–2234 (2014).
3. A. Bergström, *et al.*, Grey wolf genomic history reveals a dual ancestry of dogs. *Nature* **607**, 313–320 (2022).
4. J. Plassais, *et al.*, Whole genome sequencing of canids reveals genomic regions under selection and variants influencing morphology. *Nat. Commun.* **10**, 1489 (2019).
5. Y.-H. Liu, *et al.*, Whole-genome sequencing of African dogs provides insights into adaptations against tropical parasites. *Mol. Biol. Evol.* **35**, 287–298 (2018).
6. M. Kardos, *et al.*, Genomic consequences of intensive inbreeding in an isolated wolf population. *Nat. Ecol. Evol.* **2**, 124–131 (2018).
7. M.-H. S. Sinding, *et al.*, Population genomics of grey wolves and wolf-like canids in North America. *PLoS Genet.* **14**, e1007745 (2018).
8. S. Gopalakrishnan, *et al.*, Interspecific gene flow shaped the evolution of the genus Canis. *Curr. Biol.* **28**, 3441–3449.e5 (2018).
9. A. Bergström, *et al.*, Origins and genetic legacy of prehistoric dogs. *Science* **370**, 557–564 (2020).
10. P. Skoglund, E. Ersmark, E. Palkopoulou, L. Dalén, Ancient wolf genome reveals an early divergence of domestic dog ancestors and admixture into high-latitude breeds. *Curr. Biol.* **25**, 1515–1519 (2015).
11. L. A. F. Frantz, *et al.*, Genomic and archaeological evidence suggest a dual origin of domestic dogs. *Science* **352**, 1228–1231 (2016).
12. L. R. Botigué, *et al.*, Ancient European dog genomes reveal continuity since the Early Neolithic. *Nat. Commun.* **8**, 16082 (2017).
13. M. Ní Leathlobhair, *et al.*, The evolutionary history of dogs in the Americas. *Science* **361**, 81–85 (2018).
14. M.-H. S. Sinding, *et al.*, Arctic-adapted dogs emerged at the Pleistocene–Holocene transition. *Science* **368**, 1495–1499 (2020).
15. J. Ramos-Madrigal, *et al.*, Genomes of Pleistocene Siberian wolves uncover multiple extinct wolf lineages. *Curr. Biol.* **31**, 198–206.e8 (2021).
16. D. H. Alexander, J. Novembre, K. Lange, Fast model-based estimation of ancestry in unrelated individuals. *Genome Res.* **19**, 1655–1664 (2009).
17. W. Haak, *et al.*, Massive migration from the steppe was a source for Indo-European languages in Europe. *Nature* **522**, 207 (2015).
18. N. Patterson, *et al.*, Ancient admixture in human history. *Genetics* **192**, 1065–1093 (2012).
19. J. Storå, *Reading Bones. Stone Age Hunters and Seals in the Baltic. Stockholm Studies in Archaeology 21* (2001).
20. J. Ekman, Djurbensmaterialet från stenålderslokalen Ire. Hangvar sn, Gotland. *Janzon, G. Gotlands mellanneolitiska gravar. Acta Universitatis Stockholmiensis. Studies in North-European Archaeology* **6**, 212–246 (1974).
21. M. Germonpré, E.-L. Jimenez, M. Boudin, A Late Glacial Palaeolithic Dog from Goyet (third cave, bone level A1), Belgium. [Preprint] (2021). Available at: http://dx.doi.org/10.11588/PROPYLAEUM.950.C12576.

22. A. Pira, On bone deposits in the cave “stora förvar” on the isle of stora karlsö, Sweden. *Acta Zool.* **7**, 123–217 (1926).
23. A. T. Peterson, Uses and requirements of ecological niche models and related distributional models. *Biodivers. Inf.* **3**, 59–72 (2006).
24. G. Eriksson, Part-time farmers or hard-core sealers? Västerbjers studied by means of stable isotope analysis. *J. Anthropol. Archaeol.* **23**, 135–162 (2004).
25. G. Eriksson, *et al.*, Same island, different diet: Cultural evolution of food practice on Öland, Sweden, from the Mesolithic to the Roman Period. *J. Anthropol. Archaeol.* **27**, 520–543 (2008).
26. R. Maring, J. Olsen, S. H. Andersen, M. A. Mannino, *It was a dog’s breakfast! A radiocarbon and isotope-based study of dogs exploring dietary change during the Mesolithic-Neolithic transition in Denmark Archaeometry* (2024).
27. M. Kanstrup, *Studie i senneolitisk di t: Analyse af stabile kulstof- og kv lstofisotoper i skeletmateriale fra ölandske grave* (2004).
28. G. Eriksson, *et al.*, Diet and mobility among Mesolithic hunter-gatherers in Motala (Sweden) - The isotope perspective. *J. Archaeol. Sci. Rep.* **17**, 904–918 (2018).
